# Stability of Psychiatric Diagnoses in Candidates to Liver Transplantation Referred to a Consultation-Liaison Psychiatry Service

**DOI:** 10.3390/jcm8060800

**Published:** 2019-06-05

**Authors:** Giorgio Mattei, Maria Moscara, Jessica Balducci, Silvia Cavana, Melissa Cherubini, Chiara Piemonte, Silvia Ferrari, Gian Maria Galeazzi

**Affiliations:** 1 Department of Biomedical, Metabolic and Neural Sciences, University of Modena and Reggio Emilia, Via G. Campi 287, 41125 Modena, Italy; balducci.jessica@gmail.com (J.B.); silviacavana@hotmail.it (S.C.); silvia.ferrari@unimore.it (S.F.); gianmaria.galeazzi@unimore.it (G.M.G.); 2 “Marco Biagi” Department of Economics & Marco Biagi Foundation, University of Modena and Reggio Emilia, Via J. Berengario, 51, 41121 Modena, Italy; 3 Department of Mental Health, AUSL Modena, Via L.A. Muratori 201, 41124 Modena, Italy; moscaramaria@gmail.com; 4 Department of Mental Health, AUSL Reggio Emilia-IRCCS, Via G. Amendola 2, 42122 Reggio Emilia, Italy; melissa.cherubini@gmail.com; 5 Villa Igea Hospital, Via Stradella, 73, 41126 Modena, Italy; chiarapiemonte@libero.it

**Keywords:** consultation–liaison psychiatry, liver transplantation, bio-psycho-social model, diagnosis, stability

## Abstract

Objective: To investigate the stability over time of the psychiatric diagnoses among candidates to liver transplantation referred to a consultation-liaison psychiatric service. Method: Descriptive study, carried out at the Consultation-Liaison Psychiatry Service (CLPS) placed at the Modena (Italy) General University Hospital. All patients waiting for liver transplantation and repeatedly referred to the CLPS were enrolled. The observation period was from 1 January 2008 to 31 December 2013. Pearson’s coefficients were calculated to measure diagnostic stability (index referral vs. last referral). Results: One hundred patients were assessed (males 67%; mean age 53 ± 7 years old). The mean number of referrals for patients was 3 ± 2. The stability rate of psychiatric diagnosis was 64%. The following diagnoses or conditions were all significantly stable (i.e., all featured by *r* > 0.5 and *p* < 0.05): Adjustment disorder, depressive disorder, comorbid anxiety/depressive disorder, substance use disorder (including alcohol), absence of any disorder, and presence of any disorder. Conclusions: The good level of diagnostic stability displayed in the sample may be a function of the clinical and organizational “style” of the CLPS, namely the focus on identifying the prevailing personality traits, defensive mechanisms, and relational patterns.

## 1. Introduction

In Italy, as elsewhere in the world, potential candidates to liver transplantation are mandatorily assessed by a psychiatrist at least once before the formal inclusion in the waiting list [1,2]. Aim of such referral is to assess eligibility to transplantation related to potentially interfering psychosocial situations such as psychopathological symptoms or psychiatric disorders. Such conditions do not necessarily and automatically mean exclusion from transplantation procedures, but may impair the outcome of the transplantation, as well as the adherence to posttransplantation medication and required changes of lifestyle [1].

Several studies support the need to establish reliable screening strategies: These should cover for the presence of psychiatric, behavioral, and psychosocial risk factors that may impair the outcome of the transplantation, the survival of the patient as well as of the organ, the adherence to the medical prescriptions, and may determine the onset of medical-psychiatric comorbidities after surgery [3,4,5].

Psychosocial risk factors are known to be particularly frequent and/or complex among candidates to liver transplantation, both before and after surgery [5]. Some of these patients suffer from HCV-related liver failure and often present with comorbid psychiatric disorders, frequently depression [6]. Heo et al. [5] argued that the prevalence of anxiety disorders is higher before transplantation, rather than after; furthermore, such disorders are frequently associated with hepatitis and cirrhosis. Lopez-Navas et al. [7] identified depressive symptoms in the 47% of a sample made up of 70 patients waiting for liver transplantation. Furthermore, among candidates to liver transplantation, there are many patients suffering from alcohol-related liver disease. This subgroup is particularly at risk of developing depressive and anxiety disorders when compared with patients affected by other types of liver disease (i.e., nonalcoholic). Moreover, the highest risk of psychiatric disorders is reported among patients presenting concomitant HCV infection and alcohol-related liver disease [8,9,10].

Psychiatric disorders are frequent also after liver transplantation. The prevalence of depression and delirium ranges from 30% to 47.4% [11,12], while the prevalence of anxiety syndromes, acute psychosis, and posttraumatic stress disorder is 26%, 7.5%, and 6.4%, respectively [11].

In medicine, diagnostic stability is defined as the level of concordance between diagnoses established on the same patient during repeated assessments over a certain period of time. Diagnostic stability is crucial to support clinical action and prognostic definition. Diagnostic stability also positively impacts communication among health professionals and to patients and relatives. Finally, it has relevant implications on training and research [13].

To our knowledge, no study is available concerning specifically the stability of psychiatric diagnosis among candidates to liver transplantation. Therefore, the aim of the present study was to assess the stability over time of psychiatric diagnosis as a result of psychiatric referrals before and/or after liver transplantation procedures.

## 2. Materials and Methods

### 2.1. Study Features and Data Collection

This is a descriptive study. The observation period was from 1 January 2008 to 31 December 2013. Data were drawn from the service database of the Consultation–Liaison Psychiatry Service (CLPS) of the General University Hospital in Modena (Italy). In particular, we collected all referrals concerning patients of both genders, waiting for liver transplantation and repeatedly referred (i.e., more than once) to the CLPS. To be included in this study, patients should fulfill the following inclusion criteria: 1. being referred for inclusion in the waiting list for liver transplantation; 2. having an age between 18 and 70 years old; 3. being repeatedly referred (at least twice). This group of patients also constituted the cases in a previous case-control study, focusing more specifically on predictors of complexity [6]. The study was approved by the Ethical Committee of the Province of Modena and conducted according to the principles of the Declaration of Helsinki.

### 2.2. Setting

This study was carried out at the Modena CLPS. Established in 1989, the CLPS delivers psychiatric consultation and liaison activities in all different wards of the general hospital (included the accident and emergency department, and excluded the pediatric ward) [14,15]. Of the average 1200 first consultations performed annually, about 5% are dedicated to patients related to liver transplantation procedures [16,17]. Four senior consultants, all with more than 20 years of expertise in the field of consultation-liaison psychiatry, work at the CLPS on daily shifts. Because of this, clinical reassessment is not always carried out by the same consultant, though discussion of more complex cases and sharing of relevant clinical information is regularly performed.

### 2.3. Statistical Analysis

STATA 13.1 (College Station, TX, USA) was used for statistics. A descriptive analysis of the sample was provided, with a specific focus on diagnostic features. Pearson’s coefficients were calculated to measure inter-rater reliability (index referral vs. last referral). A priori fixed levels to reject null hypothesis: *p* < 0.05.

## 3. Results

Of 309 patients referred to the CLPS to be potentially included in the waiting list for liver transplantation, 100 were repeatedly referred, thus representing the sample of the present study. Of these, sixty-seven patients were males, with a mean age of 53 ± 7 years. Mean number of referrals was 3 ± 2 (range: 2–15). Forty-six patients were referred twice, 27 patients three times, 14 patients four times, 7 patients five times, and 3 patients six times. For 3 more patients, higher numbers of referrals were requested: 8, 11, and 15, respectively. Seventy-six patients were married or in a stable relationship, and 84 were employed. The majority of the sample (60 patients) had a positive psychiatric history. Thirty-one patients were taking psychotropic medications at the index referral. Further details of the sample are described elsewhere [6].

Table 1 shows the diagnoses established during the index referral and the proportion of those that were confirmed during the final referral (disaggregated by psychiatric disorder). Pearson’s coefficient is provided to test reliability between index and last referral.

A moderate and significant diagnostic stability emerged for the following diagnoses: Adjustment disorder, depressive disorder, substance use disorder, as well as for ‘any disorder’ and ‘none’. To give an example, adjustment disorder was diagnosed in 18 patients at the index referral. Of those patients, 13 still received the same diagnosis at the final referral (72% of the initial 18 patients). The correlation between index and last referral was moderate (*r* = 0.51) and highly significant (*p* < 0.01).

Discordance between index and final diagnoses is displayed in Table 2, which also provides details for the changes.

Fifty-two patients received a negative psychiatric diagnosis at the index referral but were subsequently assigned a diagnosis within the final contact with the consultation service. Details on these data are displayed in Table 3.

## 4. Discussion

In our sample, the stability rate of psychiatric diagnosis was 64%. With the noticeable exception of adjustment disorder, this finding is consistent with available literature [18,19]. One important specific feature of the sample needs to be acknowledged: While the majority of studies on diagnostic stability in psychiatry were carried out in psychiatric settings, the present study recruited in a non-psychiatric setting. In this population, it is known that comorbidity with psychiatric disorders may reach one third [20,21,22], but it is anyway far lower than in a psychiatric setting.

The exception represented by adjustment disorder may be due to the fact that, in the context of consultation-liaison psychiatry, this is sometimes only a tentative diagnosis, established when sufficient information is not available yet, particularly with respect to the patient’s previous psychopathological conditions and long-term functioning. As our data suggest, it is possible that the diagnosis of adjustment disorder is more prevalent at the index referral, but undergoes a more precise delineation during the following assessments when more accurate data on patient’s history and coping styles are gathered.

Half of the patients who received no psychiatric diagnosis at the index referral ended up with one at subsequent assessments, either before or after transplantation. This other remarkable finding may be referred, on one hand, to the usually low overall prevalence of psychiatric morbidity in this clinical population, if compared to that of a psychiatric setting. On the other hand, it may be referred to the psychopathogenic role that the transplant procedure may have in itself, considering its high health risks and impact on functionality and lifestyle: preexisting psychological vulnerability, i.e., in terms of temperament and personality, may be triggered by the severe biopsychosocial stress experienced by patients into full-blown psychiatric disorders.

Our analysis pointed out a greater diagnostic variability, or lower stability, among patients who were referred to the psychiatrist several times. In other words, the more the times a patient is referred to the consultant, the lower the diagnostic stability. This finding is inconsistent with the literature [23], indicating that increased number of psychiatric assessments is coupled with increased information gathered concerning the individual patient, therefore leading to a greater diagnostic stability.

A number of possible explanations for this finding may be listed: Firstly, the specificity of the sample. A subgroup of the patients enrolled in our sample were admitted to hospital (gastroenterological or surgical units) as they presented a high level of multimorbidity and were prescribed several medications. These features account for higher risk of developing psychiatric symptoms, as well as a rapidly fluctuating clinical course.

Secondly, the specificity of the setting. The setting of consultation-liaison psychiatry prompts the consultant to collect a large amount of very relevant information about the patient from very heterogeneous sources (e.g., ward staff, clinical file, relatives, caregiver, etc.), by means of different languages (e.g., the languages of ward staff, social workers, relatives, etc.), and on different levels of communication (e.g., verbal and nonverbal). Contextual aspects of setting as the time frame (e.g., urgent referrals) or the available locations (e.g., at bedside, in medical rooms, etc.) also impact the clinical outcomes such as diagnostic definition [17,24,25,26,27].

Thirdly, the specific purposes of the referral. The task of the consultant, in the clinical area of transplantation, is mainly to detect or exclude the presence of relevant psychopathological impairments and to describe the patient’s prevalent relational patterns and coping strategies that may support or interfere with the management of the transplant procedure. The consultant is not directly in charge of the patient, but, more properly, of the medical-surgical staff in relation to the specific patient the consultation has been requested for.

Finally, the impact of high biopsychosocial complexity. Patients waiting for liver transplantation are featured by high biopsychosocial complexity, a thick interconnection of clinical factors (i.e., multimorbidity, risk of transient or nontransient cognitive impairments due to hepatic encephalopathy, need of frequent medical and pharmacological reassessments, polypharmacotherapy, etc.) and extraclinical factors (i.e., high disability and impact on functioning, impaired family relationships especially for alcohol-related liver failure, prolonged hospitalization in a place far from home, with discomfort and high indirect costs for the patient and the family, and so forth). These different factors act reciprocally in establishing a high level of frailty [28,29,30].

In the light of the above, the study of diagnostic stability is clinically useful since it provides an indirect though very accurate measure of the effectiveness and reliability of diagnostic assessments: Diagnostic stability may be considered to be a function of both the consultant’s skills and competencies and specific clinical features of the sample. Thus, by studying it, relevant information may be gathered supporting: 1. specific training initiatives of health professionals; 2. organizational improvements, i.e., implementation of clinical interviews with psychometric or clinometric assessment tools [30]; 3. design of dedicated follow-up pathways according to different diagnostic macro-areas. Moreover, with respect to the specific setting of the present study (consultation-liaison psychiatry in the field of liver transplantation), the clinical implications of this study involve three areas: Communication with the ward staff, continuity of care, and communication between patient and relatives/caregivers. With respect to the first (communication with the ward staff), studying the diagnostic stability may increase trust and reciprocity between professionals. Furthermore, the fact that the diagnostic process is substantially stable within the CLPS favors trust and reciprocity even between the personnel working in the service. With respect to the second (continuity of care), diagnostic stability favors more targeted pharmacological interventions, especially considering the high level of multimorbidity and the need to follow strict pharmacological and nonpharmacological protocols in this population. Finally, as far as the communication between patient and relatives/caregivers is concerned, a stable diagnosis favors and fosters trust from colleagues, patient, and relatives toward the treatment proposed by the ward staff.

Some limitations of this study need to be acknowledged. First, no structured diagnostic instruments were used during the psychiatric consultation; nevertheless, the diagnostic procedure is well-established after years of extensive and intensive clinical activity of the CLPS and sufficiently reliable. Second, reassessments were not always carried out by the same consultant, due to the organization of the CLPS that includes a rotation of four senior consultants working on daily shifts; continuous clinical discussions of cases both on a daily basis and during dedicated monthly interdisciplinary meetings partially compensates for this lacking. Third, due to the retrospective nature of data (already collected and anonymized), it was not possible to calculate inter-rater reliability. Despite this, the design adopted made the study feasible and provided hints for future research; moreover, the consultants who assessed candidates to liver transplantation are all featured by long-term expertise in the field of consultation-liaison psychiatry (>20 years). We aim to overcome the main limitations of our research with future studies, currently on our research agenda.

To conclude, we hypothesize that the good level of stability displayed by our sample may be due to the clinical approach used by the consultants, focused on identifying the prevailing personality traits, defensive mechanisms, and relational patterns. Further research is needed to confirm this finding.

## Figures and Tables

**Table 1:** Number of diagnoses established at the index referral confirmed at the final referral (disaggregated by type of disorder).

Diagnosis	*N* of Index Diagnoses	*N* of Final Diagnoses	% of Index Diagnoses Confirmed at Final Referral	Pearson’s *r* Coefficient
Adjustment Disorder	18	13	72.22	0.51 ***
Depressive Disorder	11	9	81.82	0.51 **
Comorbid Anxiety/Depressive Disorder	6	4	66.67	0.52
Substance misuse (including alcohol)	10	8	80.00	0.54 **
Delirium	2	1	50.00	0.91 (n.s.)
Major Neurocognitive Disorder	1	1	100.00	-
None	52	28	53.85	0.50 **
Any disorder	48	36	75.00	0.52 **
All referrals	100	64	64.00	0.51 **

Note: ** *p* < 0.05; *** *p* < 0.01; n.s.: not significant.

**Table 2:** Type and number of diagnoses changed within the final referral.

Diagnosis	*N* of Index Diagnoses	*N* of Patients Receiving a Different Diagnosis within the Final Referral (Disaggregated by Final Diagnosis)
Adjustment Disorder	18	5: Personality Disorder (*N* = 1, 5.56%) Anxiety Disorder (*N* = 1, 5.56%) Major Neurocognitive Disorder (*N* = 1, 5.56%) None (*N* = 2, 11.11%)
Depressive Disorder	11	2: Substance misuse (including alcohol) (*N* = 1) None (*N* = 1, 9.09%)
Comorbid Anxiety/Depressive Disorder	6	2: Personality Disorder (*N* = 4, 66.67%) Delirium (*N* = 1, 16.66%)
Substance misuse (including alcohol)	10	2: Adjustment Disorder (*N* = 1, 10.00%) Comorbid Anxiety/Depressive Disorder (*N* = 1, 10.00%)
Delirium	2	1: Depressive Disorder (*N* = 1, 50.00%)
Major Neurocognitive Disorder	1	-
None	52	24 (see Table 3 for details)
Total	100	64

**Table 3:** Number of negative diagnoses at the index referral (total *N* = 52) subsequently turned into positive diagnoses (disaggregated by type of disorder).

Diagnosis	*N* of Index Diagnoses	*N* of Final Diagnoses	% of Pts Receiving a Psychiatric Diagnosis at the Final Referral/Pts not Receiving a Psychiatric Diagnosis at the Index Referral
None	52	28	53.85
Adjustment Disorder	-	10	19.23
Depressive Disorder	-	6	11.54
Comorbid Anxiety/Depressive Disorder	-	1	1.92
Substance misuse (including alcohol)	-	1	1.92
Personality Disorder	-	1	1.92
Anxiety Disorder	-	1	1.92
Delirium	-	3	5.77
Major Neurocognitive Disorder	-	1	1.92
Total	52	52	100.00
